# The long and winding road of *Ascaris* larval migration: the role of mouse models

**DOI:** 10.1017/S0031182021000366

**Published:** 2021-12

**Authors:** C. V. Holland

**Affiliations:** Department of Zoology, School of Natural Sciences, Trinity College, Dublin 2, Ireland

**Keywords:** *Ascaris lumbricoides*, *Ascaris suum*, immunology, larval migration, life cycle, liver, lung, mouse models, resistance

## Abstract

*Ascaris lumbricoides* and *Ascaris suum* are helminth parasites of humans and pigs, respectively. The life cycle of *Ascaris* sets it apart from the other soil-transmitted helminths because of its hepato-tracheal migration. Larval migration contributes to underestimated morbidity in humans and pigs. This migration, coupled with a lack of a murine model in which the *Ascaris* parasite might complete its life cycle, has undoubtedly contributed to the neglected status of the ascarid. Our knowledge of the epidemiology of adult worm infections had led us to an enhanced understanding of patterns of infection such as aggregation and predisposition; however, the mechanisms underlying these complex phenomena remain elusive. Carefully controlled experiments in defined inbred strains of mice – with enhanced recovery of larvae in tandem with measurements of cellular, histopathological and molecular processes – have greatly enhanced our knowledge of the early phase of infection, a phase crucial to the success or failure of adult worm establishment. Furthermore, the recent development of a mouse model of susceptibility and resistance, with highly consistent and diverging *Ascaris* larval burdens in the murine lungs, represents the extremes of the host phenotype displayed in the aggregated distribution of worms and provides an opportunity to explore the mechanistic basis that confers predisposition to light and heavy *Ascaris* infection. Certainly, detailed knowledge of the cellular hepatic and pulmonary responses at the molecular level can be accrued from murine models of infection and, once available, may enhance our ability to develop immunomodulatory therapies to elicit resistance to infection.

## Graphical abstract

**Figure:**
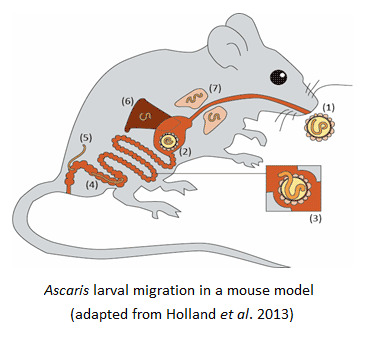

## Introduction

*Ascaris lumbricoides*, the human roundworm, and its porcine counterpart *Ascaris suum* are lumen-inhabiting adult nematode worms that produce large numbers of eggs that pass into the environment. These eggs are capable of survival under a variety of conditions, but require embryonation in order to be infective to another definitive host. After hatching from the ingested infective egg (containing an L3 larva covered by an L2 cuticle), L3 larvae migrate *via* the portal system to the liver. Some migration and growth occurs within the liver and, after that, larvae advance to the lungs, penetrate the alveolar spaces and move to the pharynx where they are coughed up and swallowed, ending up in their former location – the small intestine. This entire process takes about 67–76 days in humans (Takata, 1951). The hepato-tracheal migration sets *Ascaris* apart from the other soil-transmitted helminths, such as *Trichuris trichiura* and hookworm species, and enhances its public health significance in terms of hepatic and pulmonary morbidity (Else *et al*., 2020).

Undoubtedly, our knowledge of the properties of the adult worms and the eggs they produce is far superior to that of the tissue-resident and migrating larvae that inhabit the parenteral tissues of humans and pigs. Adult worms can be relatively easily recovered as a consequence of chemotherapeutic intervention and eggs can be obtained from either fecal or environmental material. However, our understanding of the larval migratory pathway and some of the biological properties of *Ascaris* larvae can only be obtained through *post-mortem* examination (Holland *et al*., 2013). Consequently, the public health impact of larval ascariasis remains cryptic – with the potential for liver damage in humans, being especially underestimated (Dold, 2012) – and contributes to *Ascaris* being termed one of the most neglected of the neglected tropical diseases (Holland, 2013). The logistical and ethical consequences of investigating human larval ascariasis (Stephenson, 1987; Cooper *et al*., 1992) have led to a reliance on animal models to explore the aspects of larval migration and its biological impact.

Similar to other macroparasites, one of the defining parameters that contribute to our understanding of *Ascaris* epidemiology is the intensity of infection (Walker *et al*., 2013). Macroparasite intensity is not distributed randomly among hosts – but manifests itself as an aggregated distribution (Holland *et al*., 1989; Holland and Boes, 2002) whereby few hosts carry the majority of the worms and most hosts remain uninfected or carry light infections. This phenomenon was first described by Neil Croll in Iran and heavily infected individuals were termed ‘wormy persons’ (Croll and Ghadirian, 1981); much later Poulin (2007) described aggregation as a general law of parasite ecology. Furthermore, hosts demonstrate consistency in the pattern of re-infection, termed predisposition (Elkins *et al*., 1986). An understanding of the mechanisms behind both aggregation and predisposition remains an ongoing challenge for parasitologists (Holland, 2009). One explanation for the observed epidemiological patterns may lie in the early phase of infection, and mice are ideally suited for the investigation of variation in the establishment of *Ascaris* infection and the subsequent migration of larvae through the liver and lungs.

## Animal models of ascariasis

The pig represents an obvious choice as an animal model because it is a natural host in which the entire life cycle is completed (see Roepstroff *et al*., 1997). However, pigs are expensive, require expert husbandry, have large organs for larval detection and lack versatility in terms of access to inbred strains and immunological reagents (Holland *et al*., 2013). Mice, in contrast, have a number of advantages despite the fact that the life cycle of ascarids is truncated and only encompasses the migratory phase (Lewis *et al*., 2007; Gazzinelli-Guimaraes *et al*., 2013). As our knowledge has increased, and careful comparative studies have been performed on both mice and pigs, it is clear that mice mimic the infection observed in natural hosts and can act as a suitable model for exploring the early phase of *Ascaris* infection (Murrell *et al*., 1997; Slotved *et al*., 1998). Furthermore, it is clear that this phase of infection is likely to be crucial in the manifestation of susceptibility and resistance and successful adult worm establishment (Nogueira *et al*., 2016).

All model organisms infected with *Ascaris*, other than pigs, are described as abnormal hosts. In abnormal hosts, the life cycle is incomplete and only the early stage of infection (the migratory route or pathway from the intestine *via* the liver to the lungs) can be explored. A range of abnormal hosts have been infected with *Ascaris* including mice (Stewart, 1916), rats (Stewart, 1917), guinea pigs (Douvres and Tromba, 1971), gerbils (Cho *et al*., 2007), rabbits (Galvin, 1968), cows (McCraw, 1975), lambs (Ransom and Foster, 1919) and goats (Ransom and Foster, 1919). The recovery of larvae from mice has been shown to be relatively higher than in other abnormal hosts such as rabbits, guinea pigs and rats (Douvres and Tromba, 1971) and even pigs (Roepstorff *et al*., 1997). Relative host and parasite sizes contribute to this higher recovery. It is important to emphasize that in the vast majority of these abnormal hosts, the mechanisms underlying susceptibility and resistance to ascariasis in either the liver or the lungs have not been elucidated fully (Holland *et al*., 2013).

## History of larval migration in model organisms

A series of papers on the life history and characteristics of both eggs and larvae of *A. lumbricoides* were published by Stewart (e.g. Stewart, 1916, 1917) who provided the first evidence that *Ascaris* larvae penetrate the wall of the gut and migrate to the liver and the lungs. In a detailed bulletin by Ransom and Foster (1920), that reviewed the work of Stewart and provided new data of their own, the authors concluded quite eloquently that *Ascaris* infection in rats and mice represented ‘an abortive development in animals imperfectly adapted as hosts’. It is interesting that in the early 20th century, Stewart (1917) and Ransom and Foster (1920) found evidence for pneumonia in infected rats and mice and speculated that similar pulmonary complications needed to be considered in *Ascaris*-infected children. This very early work highlights the important role that abnormal hosts can play in describing fundamental aspects of the *Ascaris* life cycle and basic parasite biology (Holland *et al*., 2013).

The seminal work of Mitchell *et al.* was the first to address the issue of resistance and susceptibility to *Ascaris* infection in a mouse model (Mitchell *et al*., 1976). These authors reported mouse strain variability in susceptibility to *Ascaris* infection utilising a range of inbred strains including C57BL/6JWehi, CBA/CaHWehi and BALB/c. However, variation in doses provided and the infectivity of different batches of eggs prevent confident direct comparison between the strains of mice used by these authors. Despite these caveats, it is interesting to note that the C57BL/6j strain emerged as the most susceptible, developing the highest larval burdens in the lungs. In resistant strains of mice, resistance was not dependent on T cells, since nude athymic strains on resistant backgrounds resisted infection just as well as the wild-type parental strains and resistance was not MHC haplotype-dependent.

Much later, Lewis *et al*. (2006) re-visited the work of Mitchell and, in the light of the developments in our understanding of the epidemiological patterns of *Ascaris* aggregation and predisposition observed in humans (Holland, 2009), developed a protocol to identify consistently susceptible and resistant strains of inbred mice to larval ascariasis (Lewis *et al*., 2006). A total of nine inbred strains of mice (A/J, BALB/c, C57BL/6j, C3H/HeN, CBA/Ca, DBA/2, NIH, SJL and SWR), utilising male mice only, were infected with a dose of 500 *A. suum* eggs (Lewis *et al*., 2006). After a preliminary experiment in which only two inbred strains were used, the numbers of larvae were enumerated on days 3–9 post-infection (p.i.) and both liver and lung tissues were investigated using a modified Baermann technique (details are described in Lewis *et al*., 2006). Three key observations were made. First, the greatest statistically significant difference in the numbers of larvae recovered from the lungs was day 7 p.i. Second, the greatest degree of accumulation of larvae was demonstrated by the C57BL/6j strain on day 7 p.i. Third, the most marked overall difference between the C57BL/6j strain and all the others was manifest in the CBA/Ca strain. Consequently, two inbred strains of mice were identified that demonstrated the most pronounced resistance/susceptibility to *Ascaris* infection. Throughout more than 10 subsequent experiments, these two strains have continued to exhibit remarkable consistency in their respective host-resistance phenotypes (Lewis, 2006; Dold, 2010; Deslyper, 2021).

The methodology employed in the study by Lewis *et al*. (2006) resulted in consistency between different experiments in worm recoveries from uniformly treated mice, and consistent between-strain variation, as well as in a significantly higher proportion of larvae being recovered compared to previous studies (Johnstone *et al*., 1978; Slotved *et al*., 1997, 1998). Furthermore, the optimum dose for such experiments was determined to be 1000 eggs but the magnitude of the dose *per se* did not influence the proportion of larvae recovered (Lewis *et al*., 2006).

## Life cycle controversies

The life cycle of *Ascaris* has, at times during the history of its investigation, proved controversial. One controversy that has now been resolved is the process of egg hatching and the identity of the larval stages that are so central to the hepato-tracheal migration. The work of Geenan *et al*. (1999) proved central to the resolution of the argument as to whether the L2 or L3 larva underwent migration to the liver, it having been generally believed earlier that the stages hatching from eggs were L2 stages (e.g. Douvres *et al*., 1969). After they described the morphology of the larvae in eggs during embryonation *in vitro* at room temperature, and subsequently detailed the infectivity of the L3 larva after oral inoculation of eggs to mice, these authors were able to conclusively demonstrate that two moults take place within the *Ascaris* egg. Geenan *et al*. emphasized that moulting to the L3 stage in ova can take place over a relatively long period of 2–6 weeks and that when using embryonated eggs to infect mice, it is important to maintain an additional maturation period in order to ensure maximum infectivity. This is borne out by the commonly-observed practice in recent studies of allowing eggs that have been dissected from worm uteri to develop for a period of 3 months to ensure full infectivity (see Oksanen *et al*., 1990).

Further important work by Fagerholm *et al*. (2000) described extensive larval growth by L3 larvae in the liver but no evidence of any further moult. These authors concluded that this retention of two moult sheaths and the migration of L3 larvae were favourable for parasite development. Furthermore, the work of Pilitt *et al*. (1981) provided a detailed description of the morphology of the L4 and L5 moults that occur after the L3 larvae return to the small intestine. After the final moult, the larvae continue growing in the small intestine as pre-adult L5 stages and then develop into the sexually mature adult male worms and females that produce eggs at patency.

## The pathological consequences of *Ascaris* larval migration in humans

Not surprisingly, our understanding of the pathological consequences of larval migration in humans remains deficient. This is partly explained by the ethical and practical factors that prevent us studying the phenomenon in human subjects. The literature on the impact of larval migration in humans remains sparse and somewhat anecdotal. A large-scale study from India investigated 510 patients over a 10-year period who were admitted to hospital with liver abscesses. Of these patients, 74 (14.5%) had biliary ascariasis as the causative agent and intact *Ascaris* were recovered from the liver abscesses in 11 patients (Javid *et al*., 1999). Among a high incidence of liver abscesses reported in children from the Western Cape in South Africa, 2% of 84 cases were attributed to *Ascaris* (Hendricks *et al*., 1997). Computerized tomography revealed hepatic lesions in three Japanese patients, all of whom had been serologically confirmed to have *A. suum* (Kakihara *et al*., 2004). Increased levels of hepatic-originating acute phase reactants – such as C-reactive protein, eosinophilic cationic protein and ferritin – were found in putatively immune Nigerian children who had been evaluated for predisposition to *A. lumbricoides* (McSharry *et al*., 1999), indicating evidence of ongoing hepatic inflammation.

Ribeiro and Fischer (2002) reviewed the various causative agents of eosinophilic lung disease (ELD). They suggest Loeffler's syndrome, first described in 1932 (Loeffler, 1932), as the first known cause of ELD. The passage of *A. lumbricoides* larvae through the lung is associated with migratory pulmonary infiltrates and raised eosinophilia (up to 70% of all white blood cells in some cases) (Loeffler, 1956). Dyspnoea and bronchospasm may be severe and Loeffler's syndrome has even been diagnosed in a neonate (Fujimura *et al*., 2001).

In an *Ascaris* mouse model, a significant reduction in body weight was observed in mice that received higher doses of *Ascaris* ova, indicating that larval migration and accumulation of larvae in the lungs has a significant impact upon host body condition even in abnormal hosts that sustain only the migratory and tissue-resident phases of infection (Lewis *et al*., 2009). Furthermore, bronchovascular damage caused by the migrating *Ascaris* larvae may result in secondary infections by opportunistic bacterial infections as observed in mice co-infected with *A. suum* and *Pastuerella multocida* in mice (Tjørnehøj *et al*., 1992). Co-morbidity of murine ascariasis and pulmonary fibrosis was recently described by Oliveira *et al*. (2019) (see also paper in this volume).

## Debate surrounding whether *A. lumbricoides* and *A. suum* represent the same or different species

Another ascarid-related debate has been whether *A. lumbricoides* and *A. suum* represent the same or different species. In an eloquent summary, Betson *et al*. (2013) concluded that based upon the biological concept of a species, the two ascarids do indeed represent separate species. Therefore, in the context of murine ascariasis, an important question is whether *A. suum* acts as an appropriate model organism for *A. lumbricoides* and whether *A. lumbricoides* would be equally tractable within a mouse model of resistance and susceptibility. It should be noted that in the vast majority of studies in which *Ascaris* species are used to infect mice (and other abnormal hosts), the species of choice tends to be *A. suum*.

In a comparative study, that to our knowledge has not been performed before, mice of both susceptible C57BL/6j and resistant CBA/Ca inbred mice were infected with either embryonated *A. suum* or *A. lumbricoides* eggs and larval counts were made from both the liver and the lungs from 6 hours to 8 days p.i. (Deslyper *et al*., 2020). The pattern of greater susceptibility on the part of the C57BL/6j strain and resistance on the part of the CBA/Ca strain was maintained for *A. lumbricoides*. However, an interesting divergence was observed between the two ascarid species: the number of *A. lumbricoides* larvae in the liver was significantly higher than those of *A. suum* and, conversely, in the lungs, the number of *A. suum* was higher than those of its human counterpart. This observation led to the hypothesis that a more pronounced immune response may be operating against *A. lumbricoides* compared to *A. suum*, especially during the liver stage. Interestingly, as long ago as in the 1950s, Sprent reported that when non-pathogen-free male white mice were infected with what was described as ‘*A. lumbricoides* from man’ or ‘*A. lumbricoides* from pig’ both species had similar migratory paths (Sprent, 1952). However, it was observed that the ‘human strain appeared to have about twice the infectivity’ with a higher number of larvae of human ascarids detected in the murine liver. In contrast to the findings of Deslyper *et al*. (2020), a higher number of larvae of the human ascarid were also observed in the lungs by Sprent (1952).

It should be noted that in the experiment by Deslyper *et al.*, *Ascaris* eggs were obtained from only two sources (*A. suum* from pigs in Belgium and *A. lumbricoides* from humans in Nigeria) and this may have limited the wider applicability of the findings (M. Betson, personal communication). A related observation by Peng *et al*. (2012) recorded that C57BL/6j mice infected with different genotypes of *Ascaris* demonstrated differences in the timing and location of egg hatching between the G1 (human) genotype and the G3 (pig) genotype, with G3 exhibiting lower hatching rates and overall larval recovery.

One relatively simple way of assessing the possible impact of immunity on parasite fitness is to measure the size of either adult worms or larvae. For example, Stewart *et al*. (1985) immunized pigs with *A. suum*, by means of repeated infection, with some pigs receiving fenbendazole treatment after each infection. After an *A. suum* challenge infection, the adult worms were measured and counted. The authors found that fewer and smaller adult worms were recovered from the groups that had received the anthelmintic after each immunising infection, compared to those that had been immunized but had not received any anthelmintic. The authors concluded that the fenbendazole treatment probably heightened the immune response against the parasite. However, an alternative explanation may be that the persistence of adult worms from the initial infections generated some local immunodepression allowing challenge worms to establish and grow better. To support this alternative hypothesis, Behnke *et al*. (1983) demonstrated, for the first time, that adult worm infection of the nematode, *Nematospiroides dubius* in mice, suppressed host immunity such that challenge infections could establish and survive.

In a comparative analysis of *A. suum* and *A. lumbricoides* in their mouse model of *Ascaris* resistance, Deslyper *et al*. (2020) also measured the larvae of the two species on days 6, 7 and 8 p.i. They made several relevant observations: first, that in mice larvae grow over time (as also observed by Lewis *et al*., 2006 among others); second, that the mean length of *A. suum* was longer than *A. lumbricoides* in both mouse strains; and third, that on day 8 p.i. (when statistical analysis was possible) the mean length of *A. lumbricoides* was significantly lower in CBA/Ca mice compared to C57Bl/6j mice, a finding that has not been observed for *A. suum*.

Two studies that add weight to the conclusion that enhanced immunity impacts upon larval size include those of Johnstone *et al*. (1978) and Song *et al*. (1985). Male C57BL/6 mice were orally infected with *A. suum*, followed by a challenge infection (Johnstone *et al*., 1978). The larvae recovered from these mice were compared to mice that had received one single dose of eggs. The authors found that hepatic larval counts were quite similar; however, the difference in larval counts was quite substantial in the lungs, with the non-immunized mice having a much higher larval count compared to that in the immunized animals. These authors, therefore, confirm the idea that ‘the mechanism of immunity against *A. suum* operates primarily in the liver rather than in the gastrointestinal tract’ (Johnstone *et al*., 1978). Between days 5 and 9 p.i., the difference in larval lengths in the liver was significantly lower for the immunized animals compared to non-immunized animals suggesting that larvae grew slower in immunized mice, compared with non-immunized controls. So, despite there being no significant difference in larval burdens in the liver, the larvae were already smaller at this time. This trend continued in the lungs, where a statistically significant difference in larval lengths was observed.

The experiment by Song *et al*. (1985), comparing primary *A. suum*-infected and reinfected mice (no description provided) demonstrated that larval length in both the liver and lungs was lower for the reinfected mice. Furthermore, for liver-inhabiting larvae, the difference in larval length was relatively small during early infection, but it increased over time. In contrast, for lung-inhabiting larvae, the initial differences were quite big, but the difference actually decreased with time. The authors concluded that the ‘development of larvae in the liver of immune mice were probably repressed by the immune mechanisms being rised [sic] in the livers’ (Song *et al*., 1985).

## Hepatic ascariasis in mice

In general, the hepatic phase of larval ascariasis has received less attention despite the acknowledgement that the liver is likely to be a key organ both in terms of its immunotolerant properties but also as an organ of larval attrition (Deslyper *et al*., 2019*a*). However, the liver has been consistently identified as a key site in the immobilization of migrating *Ascaris* larvae in mice (Sprent and Chen, 1949; Taffs, 1968; Mitchell *et al*., 1976; Johnstone *et al*., 1978; Song *et al*., 1985). In the mouse model of susceptibility and resistance, the hepatic histopathological inflammatory response was compared in C57BL/6j and CBA/Ca mice and revealed an important difference in the timing of the response between the two strains (Dold *et al*., 2010). Specifically, in resistant CBA mice, the most pronounced response occurred on day 4 of infection, which coincides with the migration of lower larval numbers from the liver to the lungs. In contrast, the severe inflammatory response in C57BL/6j mice took place on day 6 p.i., a time by which most larvae have already, successfully migrated to the lungs. Significantly, in immunized guinea pigs, day 4 p.i. had been previously identified as an important time point in the encapsulation of larvae by inflammatory cells, with an associated decrease in larval burden in the lungs (Soulsby, 1957; Khoury *et al*., 1977). Therefore, these data suggest a possible inflammatory mechanism of resistance to larval *Ascaris* migration, acting rapidly and focused in the liver.

Deslyper *et al*. (2016) sought to identify changes in protein expression potentially linked to both resistance and susceptibility amongst the two strains and for this used high-throughput quantitative mass spectrometry to facilitate a proteomic approach. They analysed the hepatic proteomes of both *A. suum*-infected and control C57BL/6J and CBA/Ca mice on day 4 p.i. This day was chosen because of the earlier observations of Dold *et al*. (2010) that identified day 4 as a key day in terms of the enhanced histopathological response observed in CBA/Ca mice. Over 3000 proteins were identified, and clear intrinsic differences were elucidated between the two strains. These included a higher abundance of mitochondrial proteins, particularly those associated with the oxidative phosphorylation pathway and reactive oxygen species (ROS) production, in the relatively resistant CBA/Ca mice. The authors hypothesized that the increased ROS levels associated with higher levels of mitochondrial activity result in a highly oxidative cellular environment that has a dramatic effect on the nematode's ability to successfully sustain a parasitic association with its resistant host. Both infected strains had increased abundances in proteins associated with the oxidative phosphorylation pathway, as well as the tricarboxylic acid cycle, with respect to their controls, indicating a general stress response to *Ascaris* infection.

Despite the early stage of infection, some immune-associated proteins were found to be differentially abundant. In general, the susceptible C57BL/6j mice displayed higher abundances of immune-associated proteins compared to the resistant CBA/Ca mice. The complement component C8a and S100 proteins, S100a8 and S100a9 that can be produced alongside complement proteins, were highly differentially abundant in both infected strains, signifying a potential innate immune response and the importance of the complement pathway in defence against macroparasite infection. In addition, the signatures of an early adaptive immune response were observed through the presence of proteins, such as plastin-2 and dipeptidyl peptidase 1. A marked decrease in proteins associated with translation was also observed in both infected strains, indicative of either a general response to *Ascaris* or a modulatory effect by the nematode itself.

Building upon this work, Deslyper *et al*. (2019*b*) also implemented the proteomic approach on day 7 p.i. This is the day when larval numbers peak in the murine lung in this model system and the divergence between the strains is most pronounced. Hence, it was anticipated that a stronger immune signature would be apparent on this later date.

Their earlier observations concerning a higher abundance of proteins involved in oxidative phosphorylation and associated mitochondrial proteins in CBA/Ca mice were confirmed. Novel observations included a differential response between the two mouse strains with respect to the initiation of the complement system, an important first step in the activation of the innate immune response. The CBA/Ca strain displayed an increased abundance in proteins involved in the lectin pathway of the complement system; specifically, with both Mbl1 and Mbl2 being significantly upregulated in infected CBA/Ca when compared to infected C57BL/6J mice. Conversely, infected C57BL/6J had more upregulated proteins involved with complement inhibition, Cfi and Cfh in particular, when compared to its CBA/Ca-infected counterpart. Additionally, Cfb was upregulated in C57BL/6J-infected mice compared to their uninfected controls. This suggested a downregulation of complement activation in the C57BL/6J strain and an upregulation of the lectin pathway in the CBA/Ca strain. The authors concluded that the ability of *Ascaris* to modulate the host response is particularly mouse strain-dependant. However, the mechanisms of how *Ascaris* evades or suppresses the immune system and the factors involved in immunomodulation have yet to be determined (see Deslyper *et al*., 2021 below).

Another novel finding was the observation that retinol metabolism was increased in C57Bl/6J mice. This is particularly intriguing because the relationship between *Ascaris* infection and vitamin A status has been a focus in human studies (Taren *et al*., 1987; Al-Mekhlafi *et al*., 2014). C57BL/6J mice have a higher abundance of proteins involved in retinol metabolism when compared to CBA/Ca, irrespective of infection status. Furthermore, infected C57BL/6J mice have a higher abundance of these proteins when compared to their controls. Infected C57BL/6J mice exhibited a higher abundance of Cyp3A and Cyp1A proteins compared to infected CBA/Ca mice. These proteins degrade retinoic acid (known as atRA) and, consequently, could play a role in reducing its transcriptional function (Nebert and Russell, 2002). Pigs infected with *A. suum* were fed atRA and demonstrated an increased abundance of hepatic mRNA of Th2-associated cytokines (Dawson *et al*., 2009). The presence of atRA might, therefore, indicate an early shift to the Th2 response as outlined by Pino-Lagos *et al*. (2008).

Furthermore, ABA-1, an allergen found in somatic tissues of *Ascaris* and secreted by both larval and adult stages of the parasite, has been found to bind retinol among other substances such as fatty acids (Xia *et al*., 2000). This could indicate a regulatory mechanism by the parasite on retinol levels in the host and, consequently, contribute to the control of the Th2 response mounted by the host. These results confirm a potential role for retinol and its metabolites during *Ascaris* infection in the liver. However, it is unclear what exactly this role may be and how retinol metabolism might influence the hosts' response.

Following on from the proteomic work that focused upon *A. suum* only (Deslyper *et al*., 2016, 2019*b*) and the quantitative comparative studies on the larval migration of *A. suum* and *A. lumbricoides* (Deslyper *et al*., 2020), it was concluded that it was important, as a next step, to determine which immune cells are activated in the liver and whether these cells differed between the two strains of mice and the two species of ascarid. Therefore, both susceptible and resistant strains of mice were infected with *A. suum* (the porcine ascarid) and *A. lumbricoides* (the human ascarid) and immune cells in their livers and spleens were enumerated using flow cytometry (Deslyper *et al*., 2021). Day 7 p.i. was selected because of evidence for an altered hepatic immune response between the two mouse strains during *A. suum* infection had been observed at this stage (Deslyper *et al*., 2019*b*).

In summary, the influence of mouse strain on the generated immune cells was greater than that of parasite species with *A. suum* and *A. lumbricoides* exhibiting qualitatively similar effects on splenic and hepatic myeloid and lymphoid cell repertoires. Infection with either ascarid species resulted in increases in the numbers of myeloid cells, including monocytes, dendritic cells, Kupffer cells and eosinophils in the livers, and these cells are likely to contribute to parasite elimination. Unexpectedly, the numbers of all hepatic lymphocyte subsets including *αβ* T cells, *γδ* T cells, B cells, natural killer cells and natural killer T cells increased in the susceptible C57BL/6J mice, but decreased in the relatively resistant CBA/Ca mice after infection with either parasite species. This suggests that the susceptible mice mounted more robust immune responses to the larvae. This mirrors observations from the proteomic analyses that susceptible C57BL/6J mice displayed higher abundances in immune-associated proteins compared to the resistant CBA/Ca mice (Deslyper *et al*., 2016). This might be explained by the responses being more tolerogenic in C57BL/6J mice, leading to higher subsequent larval burdens in the lungs. Liver dendritic cells, Kupffer cells and monocytes are well-documented to preferentially induce tolerance over immunity to antigens encountered in the liver (Doherty, 2016). Alternatively, the livers of the susceptible C57BL/6J mice may receive higher numbers of larvae producing stimulatory antigens, requiring a more robust hepatic immune response compared with that of CBA/Ca mice. Uninfected and *Ascaris*-infected CBA/Ca mice had higher numbers of CD4+ T cells and *γδ* T cells in their spleens, which might arise from mesenteric lymph node activation by parasite antigens. Future functional studies are needed to elucidate the immunogenic *vs* tolerogenic roles of hepatic leucocytes in *Ascaris* infection.

## Pulmonary ascariasis in mice

The passage of *Ascaris* larvae through the lungs of both animal models and humans is known to contribute to significant respiratory distress (Matsuyama *et al*., 1998; Nogueira *et al*., 2016). However, the role of pulmonary inflammatory responses in primary infections remains unclear (Lewis *et al*., 2007). Quantitative studies of the larval burden of *Ascaris* in both liver and lungs confirm the lungs as the organ of accumulation, with the numbers of larvae peaking at day 7 p.i. (Lewis *et al*., 2006).

The significance of inflammatory processes within the lungs of C57BL/6J and CBA/Ca mice was explored using the leucocyte population in bronchoalveolar lavage (BAL) fluid and lung histopathology (Lewis *et al*., 2007). The susceptible mice produced a BAL response almost twice as intense as that of resistant mice – with stronger neutrophil, lymphocyte and eosinophil but not macrophage components – suggesting that the difference in larval burdens between the two strains was generated earlier in the course of infection. These results were further corroborated by a histological examination of the lung tissues which showed that the passage of the larval stages of *A. suum* through the mouse lungs was associated with a marked inflammatory response in both strains. Again, C57BL/6J mice exhibited increased inflammation relative to CBA/Ca mice. The authors concluded that the responses mirrored larval intensity and that the pulmonary inflammatory immune response was not prominently involved in the primary protection of mice to *Ascaris* infection in the later days of infection in the lungs. The lack of support for a pulmonary mechanism led to the suggestion that a hepatic/post-hepatic factor that varies between C57BL/6J and CBA/Ca mice may play a critical role in the successful migration through host tissues.

In the first of two informative papers, Gazzinelli-Guimaraes *et al*. (2013) initially explored histopathology and cellular and humoral responses in the lungs of BALB/c mice singly infected with *A. suum*. A polarized pro-inflammatory innate response was observed with increases in IL-6, TNF-*α* and IL-5. A significant correlation was observed between IL-6 production and the numbers of larvae in the lungs of the mice. Furthermore, histopathology revealed an increase in inflammatory infiltrate in infected mice with many eosinophils observed on day 12 p.i. This finding of enhanced eosinophil numbers in the lungs of *A. suum*-infected BALB/c mice was also observed by Enobe *et al*. (2006), peaking at day 14 p.i. Gazzinelli-Guimaraes *et al*. (2013) also commented upon the likelihood of a switch from this pro-inflammatory response observed in a mouse model of early infection, to a Th2 response observed in *Ascaris*-infected humans in endemic areas (Cooper *et al*., 2000). They postulated that this switch could occur ‘at the end of the tissue migratory phase or after adult worms have reached patency in the intestine’ (Gazzinelli-Guimaraes *et al*., 2013). The study also briefly addressed the question of the use of *A. lumbricoides* in a mouse model. However, comparing only lung samples derived from day 8 p.i., no significant difference between *A. suum* and *A. lumbricoides* larval burdens was detected.

Following on from this work, Nogueira *et al*. (2016) performed a detailed comparative analysis on single and multiple *A. suum*-infected BALB/c mice, undertaking larval counts, histopathology of lung tissue, BAL and flow cytometry to detect systemic cytokines and even an assessment of lung function. The authors observed a significant reduction in larval burden in the lung, but not the liver, of reinfected mice. Multiple exposure led to an increase in circulatory inflammatory cells and the production of systemic cytokines such as IL-4, IL-5, IL-6, IL-10, Il-17A and TNF-*α*. They concluded that ‘a chronic and robust immune response was observed in the lungs of reinfected mice leading to tissue damage and protection against progression of the parasite cycle’ (Nogueira *et al*., 2016). Importantly, as an insight into the public health significance of larval ascariasis, multiply-exposed mice manifested altered lung function as a consequence of lung injury and the inflammatory immune response to infection. The role of inflammation in the induction of protection against helminth infection during pulmonary migration, albeit with accompanying respiratory malfunction, is discussed. These observations in mice may provide some explanation for the enhanced indicators of inflammation observed in putatively immune (‘probably multiple exposed’) *vs* susceptible (‘likely to suffer first exposure’) Nigerian children whose predisposition status had been assessed by multiple worm burden quantification (McSharry *et al*., 1999; words in brackets provided by Nogueira *et al*., 2016). Further studies are required to elucidate the mechanisms behind these important observations and mouse models provide the ideal system within which to dissect these complex interactions between larval migration in both the liver and the lungs, and the consequent immunological and pathological outcomes.

## Future directions

Evidence has accumulated to confirm that selective phases of *Ascaris* infection and their associated host response are tractable within murine models of ascariasis. Given the differential hepatic inflammatory response observed between susceptible and resistant strains of mice, questions can be raised as to what drives the innate immune response and the spectrum of secreted cytokines, and consequently activated effector cells, that contribute to host resistance. Preliminary work on the hepatic immune response suggests that strain differences in hosts are more important than differential responses to *Ascaris* species (Deslyper *et al*., 2021), despite the differences observed in terms of larval accumulation in the lungs (Deslyper *et al*., 2020). However, Nejsum *et al*. (2008) infected pigs with different genotypes of *A. suum* (unique mtDNA haplotypes) and observed an uneven distribution of different genotypes within the host intestine. These observations suggest that introducing parasite genetic variation within a mouse model of ascariasis could be highly informative and would require careful elucidation. The complex results that arose from the experiments on hepatic immunity (Deslyper *et al*., 2021) merit repetition with larger numbers of mice and at more days post-infection. As stated by Nogueira *et al*. (2016), further work is required to elucidate the immune functions, mechanisms and pathways of protection that are triggered to control larval ascariasis burden and tissue damage.

Other avenues of research can take advantage of a model of early *Ascaris* infection in mice. Among naturally-infected hosts, co-infection is the norm (Vaumourin *et al*., 2015). Several other important microparasite and macroparasite infections utilize the liver as a site of infection or spend part of their life-cycle therein, such as *Schistosoma* and *Plasmodium* (Deslyper *et al*., 2019*a*). Hepatitis B and *Mycobacterium tuberculosis* are potentially important co-infections that inhabit the liver and lungs, respectively (Abanyie and Lamb, 2013). Pre-existing immune responses to these other pathogens may impact upon larval migration both in terms of enhancement or diminution of the accompanying host responses and concurrent parasite burdens. Pigs coinfected with *Entamoeba coli* and lung-stage *Ascaris* developed more severe lung pathology and bacteria were carried to the lungs by the migrating *Ascaris* larvae (Adedeji *et al*., 1989).

In a thought-provoking article, Midha *et al*. (2021) discuss how *Ascaris* may interact with the microbiome, either during larval migration or in its final location in the host intestine. Immune and endothelial cells in the gut, portal system, liver and lung are exposed to bacterial components or live microbes during larval migration. The authors pose the question as to whether certain bacterial species influence tissue migration, a hypothesis that could be explored in a mouse model.

In a genetic experiment that was performed in order to explore the influence of the Intelectin-2 gene (Itln-2 has been found to have an anti-nematode role – Pemberton *et al*., 2004) on susceptibility to *Ascaris* in a mouse model (Dold *et al*., 2011); a pilot experiment utilising male and female mice of both CBA and C57BL/6J strains in a 1:1 ratio indicated that mouse sex did not have a significant influence on larval burden, although male C57BL/6 mice did have arithmetically higher mean worm burdens than female mice of the same strain. However, in a backcross experiment that utilized male and female mice in a 1:3 ratio, male mice of the susceptible strain, C57Bl/6J, manifested much higher larval burdens in their lungs than female mice. However, the male and female mice of the resistant CBA strain had very similar larval burdens and hence no sex bias in this strain of mice was evident. Therefore, there was evidence of enhanced susceptibility of male C57Bl/6J mice.

The mechanism behind these differences in larval burden requires further investigation but, in contrast to earlier work based on male mice only, the backcross experiment required mice of both sexes to be in close proximity in the animal house, albeit in separate cages. In captivity, aggression in male rodents can be triggered by female scent alone (Barnard *et al*., 1997). Therefore, the scent of the females in close proximity may have increased aggression and investment in courtship behaviours among the male mice. Dominance hierarchies have been documented previously in response to the protozoan *Babesia microti* (Barnard *et al*., 1994). Reduced resistance among high-ranking males was associated with increased serum testosterone and corticosterone concentration and reduced serum immunoglobulin. Endogenous sex steroid hormones have been found to have a direct effect on the generation of a Th2 response to *T. muris* (Hepworth *et al*., 2010).

The dramatically increased larval burdens in a number of male C57BL/6J mice is of particular interest as previous attempts at immunosuppression, using the steroid hydrocortisone, did not enhance *A. suum* larval burdens in the susceptible mouse strain (Lewis *et al*., 2007). Therefore, it is possible that the stressor resulting from the presence of females in close proximity induced heightened testosterone levels in male individuals, which lead to some form of potent immunosuppression. The contrasting results between work conducted by Lewis *et al*. (2007) and Dold *et al*. (2011) may indicate that a single pulse administration of hydrocortisone does not have as great an influence on the mechanism of resistance as a continuous stressor.

## Conclusion

The infection of mice with *Ascaris* has had a long history and has provided key pieces of evidence that have illuminated aspects of the biology, life cycle and larval migration of this neglected ascarid. Furthermore, we still require the versatility that murine models confer on our ability to model the complexity of parasite life cycles and their epidemiology.

Undoubtedly, other soil-transmitted helminths, such as *T. trichiura* and hookworm species, possess murine equivalents. These include *Trichuris muris* (Cliffe and Grencis, 2004) and *Heligmosomoides bakeri* (Behnke *et al*., 2009) that complete their life-cycles in mice. In a yet to be repeated experiment that mimicked natural conditions, Tanguay and Scott (1992) infected mice with *H. bakeri* and explored the contribution of host genetics, behaviour and acquired resistance to the observed aggregation. The lack of such a murine model system in which *Ascaris* could complete its life cycle has undoubtedly contributed to its neglected status (Holland *et al*., 2013). However, as concluded by Dold *et al*. (2010), the two mouse strains, C57BL/6J and CBA/Ca, with the highly consistent and diverging *Ascaris* larval burdens in their lungs, represent the extremes of the host phenotype displayed in the aggregated distribution and provide an opportunity to explore the mechanistic basis that confers resistance and predisposition to light and heavy *Ascaris* infection.

Certainly, detailed knowledge of the cellular hepatic and pulmonary response at the molecular level could be made available from murine models of infection and, once available, may enhance our ability to develop immunomodulatory therapies to elicit resistance to infection in both people and pigs. More generally, increasing our knowledge of larval ascariasis will contribute to the development of intervention strategies for early stages of the life cycle, aimed at preventing parasite establishment and thus chronic disease.
